# Iron starvation results in up-regulation of a probable *Haloferax volcanii* siderophore transporter

**DOI:** 10.3389/fmicb.2024.1422844

**Published:** 2024-08-14

**Authors:** Anna-Lena Sailer, Zivojin Jevtic, Britta Stoll, Julia Wörtz, Kundan Sharma, Henning Urlaub, Mike Dyall-Smith, Friedhelm Pfeiffer, Anita Marchfelder, Christof Lenz

**Affiliations:** ^1^Biology II, Ulm University, Ulm, Germany; ^2^Department of Biomedicine, University Children’s Hospital, University of Basel, Basel, Switzerland; ^3^Department of Biomedicine, University of Basel, Basel, Switzerland; ^4^Bioanalytical Mass Spectrometry Group, Max Planck Institute for Multidisciplinary Sciences, Göttingen, Germany; ^5^Bioanalytics Group, Department of Clinical Chemistry, University Medical Center Göttingen, Göttingen, Germany; ^6^Computational Systems Biochemistry, Max Planck Institute for Biochemistry, Martinsried, Germany; ^7^Veterinary Biosciences, Faculty of Science, Melbourne Veterinary School, University of Melbourne, Parkville, VIC, Australia

**Keywords:** *Haloferax volcanii*, proteome, iron starvation, data-independent acquisition mass spectrometry, DIA-MS, import/export processes, metal homeostasis

## Abstract

The response of the haloarchaeal model organism *Haloferax volcanii* to iron starvation was analyzed at the proteome level by data-independent acquisition mass spectrometry. Cells grown in minimal medium with normal iron levels were compared to those grown under low iron conditions, with samples being separated into membrane and cytoplasmic fractions in order to focus on import/export processes which are frequently associated with metal homeostasis. Iron starvation not only caused a severe retardation of growth but also altered the levels of many proteins. Using a comprehensive annotated spectral library and data-independent acquisition mass spectrometry (DIA-MS), we found that iron starvation resulted in significant changes to both the membrane and the soluble proteomes of *Hfx. volcanii*. The most affected protein is the RND family permease HVO_A0467, which is 44-fold enriched in cells grown under iron starvation. The gene HVO_A0467 can be deleted suggesting that it is not essential under standard conditions. Compared to wild type cells the deletion strain shows only slight changes in growth and cell morphologies show no differences. Molecular docking predictions indicated that HVO_A0467 may be an exporter of the siderophore schizokinen for which a potential biosynthesis cluster is encoded in the *Hfx. volcanii* genome. Together, these findings confirm the importance of iron for archaeal cells and suggest HVO_0467 as a siderophore exporter.

## 1 Introduction

Despite being one of the most frequent elements on earth, the bioavailability of iron is often limited in natural environments due to the low solubility of ferric iron (Fe^3+^) which is the predominant oxidation state in oxic environments. Iron is an essential element for most microbial organisms due to its key role in, e.g., iron–sulfur clusters, iron centers, and heme groups. Consequently, organisms have developed various iron-acquisition pathways, among others high-affinity iron uptake systems which often use highly specific iron-binding compounds, i.e., siderophores (Miethke and Marahiel, 2007). Understanding the response of organisms toward iron limitation conditions (hereafter referred to as iron starvation) provides insight into how they have adapted to cope with stresses in their natural environment.

A lot of attention has been devoted to the response mechanisms of bacteria to iron starvation, including changes in the biosynthesis, secretion and uptake of siderophores to sequester iron. Surprisingly little is known, however, about the iron starvation response of archaea, e.g., halophilic archaea (here referred to as haloarchaea). These commonly live in poorly oxygenated environments at extremely high salt concentrations. Several studies have dealt with iron biology of haloarchaea, mainly using the model species *Haloferax volcanii* and *Halobacterium salinarum*. The effect of iron limitation on the respiratory chain of *Hbt. salinarum* has been analyzed (Hubmacher et al., 2003). Iron uptake has been measured in *Hbt. salinarum* strain JW5 and was attributed to an energy-dependent iron uptake process (Hubmacher et al., 2007). However, the responsible gene has not yet been identified. In *Halobacterium*, the SirR/DtxR family transcription regulators Idr1 and Idr2 were characterized and shown to be involved in iron homeostasis (Schmid et al., 2011).

In *Hfx. volcanii*, the genes *iucA* and *iucC* were identified by the antiSMASH server as predicting the synthesis of the siderophore schizokinen (Blin et al., 2023). A detailed bioinformatic reconstruction (Niessen and Soppa, 2020) identified a six-gene cluster which codes for a siderophore biosynthetic pathway. The products of four genes (*iucABDC*) were identified by bioinformatics as converting the precursor diaminopropane to schizokinen and the other two genes (*dat*, *bdb*) as specifying enyzmes that generate diaminopropane from aspartate-semialdehyde. The transcription of the cluster is induced under iron limitation conditions and deletion of the *iucABDC* genes resulted in a growth defect at very low concentrations of Fe^3+^ but not of Fe^2+^ (Niessen and Soppa, 2020). This is consistent with schizokinen having an extremely high affinity for Fe^3+^ (Mullis et al., 1971; Storey et al., 2006; Chuljerm et al., 2019, 2020). More recently, three diphtheria toxin repressor proteins Idr, SirR, and TroR were identified as potential transcriptional regulators of iron homeostasis (Martinez Pastor et al., 2024).

*Hfx. volcanii* is a model organism for halophilic archaea, the genome is sequenced (Hartman et al., 2010) and its biology is under intense study (Haque et al., 2020), leading to increased functional annotation and understanding. One key method to study the response of an organism to different environmental conditions is quantitative proteomics. Several proteomic analyses of *Hfx. volcanii* have been reported, such as the response to oxidative stress induced by hypochlorite (McMillan et al., 2018), changes in salt concentration and temperature (Jevtic et al., 2019), and the transition from exponential to stationary phase (Cerletti et al., 2018). Multiple proteome data sets available for *Hfx. volcanii* have been analyzed in a community effort and combined in ArcPP, the archaeal proteome project (Schulze et al., 2020). None of these global data sets, however, included *Hfx. volcanii* cultivated under conditions of iron starvation. We therefore set out to perform a comprehensive proteome study under these conditions using label-free data-independent acquisition mass spectrometry (DIA-MS). DIA-MS is a well-established method that provides high coverage of identified and quantified proteins without disturbing the experimental system. In addition, we selected to study the impact of iron starvation on membrane-associated and soluble proteins separately following fractionation, since many known iron scavenging and import processes in other species rely on dedicated transmembrane import systems. We therefore chose a fractionated approach to obtain higher coverage of membrane-associated proteins, and closely interrogated the data both for general stress response mechanisms and, more specifically, for evidence of iron uptake mechanisms. Furthermore, we investigated the proteins most affected under iron starvation in more detail.

## 2 Materials and methods

### 2.1 Strains and growth conditions

*Hfx. volcanii* strain H119 was used as wild type strain for all experiments, as reported in a previous publication (Jevtic et al., 2019). In short, it is derived from wild-type strain DS2 by removal of plasmid pHV2 and deletion of three genes that can be used as selection markers (Δ*pyrE2*, Δ*trpA*, Δ*leuB*). H119 was grown aerobically with shaking (200 rpm) to exponential phase (0.55 OD_650_) at 45°C in 2.2 M salt in minimal medium Hv-min (Allers et al., 2004) containing normal or low iron (normal: 0.0083 mM FeSO_4_*7H_2_O; low iron: FeSO_4_*7H_2_O was omitted; no efforts to further minimize residual iron were taken). Two biological replicates were prepared for each sample. In an additional control experiment, cells were grown in complex medium Hv-YPC (Allers et al., 2004) at three biological replicates per sample. Refer to Supplementary Table S3 for an overview of the samples.

### 2.2 Protein isolation and in-solution digestion

Cells were harvested by centrifugation, resuspended in 18% saltwater (2.46 M NaCl, 88 mM MgCl_2_, 85 mM MgSO_4_, 56 mM KCl, 12 mM Tris–HCl, pH 7.5), sonicated and subsequently incubated with sodium taurodeoxycholate to a final concentration of 0.006% as reported previously (Jevtic et al., 2019). Membranes were collected by centrifugation (100,000 × g for 1 h at 4°C), and membrane pellets and supernatants were processed separately. Membranes were resuspended by sonication in 5 mL 18% saltwater. Both samples were incubated with DNAse I, exonuclease III, and RNase A to digest nucleic acids. Aliquots of 0.5 mL were frozen in liquid nitrogen and stored at −80°C. Aliquots were thawed and proteins were precipitated by adding 4.5 mL 100% cold acetone and incubation at −20°C overnight. Proteins were pelleted by centrifugation at 6,000 × g for 60 min at 4°C. Pellets were washed four to five times with 80% acetone and in a final washing step with 5 mL 80% ethanol. Pellets were air-dried and frozen in liquid nitrogen until further use.

Forty micrograms of precipitated protein per biological replicate were solubilized using 3-[(2-methyl-2-undecyl-1,3-dioxolan-4-yl)methoxy]-1-propanesulfonate cleavable surfactant (Rapigest, Waters). Following reduction and alkylation with dithiothreitol and iodoacetamide, proteins were digested using sequencing grade porcine trypsin (Promega) at an enzyme-to-substrate ratio of 1:50 (w:w). Following acidic cleavage of the surfactant, the resulting fatty acids were pelleted by centrifugation and removed. Supernatant peptide mixtures were dried in a SpeedVac concentrator and stored at −20°C prior to analysis.

### 2.3 Mass spectrometry acquisition

The mass spectrometric analysis was performed as described previously (Jevtic et al., 2019). For generation of an annotated MS/MS spectral library, peptide aliquots of three biological replicates of a *Hfx. volcanii* culture grown in complex medium were pooled to a total amount of 80 μg, and separated into eight fractions using a reversed phase spin column (Pierce High pH Reversed-Phase Peptide Fractionation Kit, Thermo Fisher Scientific). These fractions, as well as all biological replicates of membrane and soluble samples, were spiked with a synthetic peptide standard (iRT Standard, Biognosys) for retention time alignment.

Samples were analyzed on a nanoflow chromatography system (Eksigent nanoLC425) hyphenated to a hybrid triple quadrupole-TOF mass spectrometer (TripleTOF 5,600+) equipped with a Nanospray III ion source (Ionspray Voltage 2,400 V, Interface Heater Temperature 150°C, Sheath Gas Setting 12) and controlled by Analyst TF 1.7.1 software build 1,163 (all Sciex). In brief, peptides were dissolved in loading buffer (2% acetonitrile, 0.1% formic acid v/v in water) to a concentration of 0.3 μg/μL. For each analysis, 1.5 μg of digested protein was enriched on a precolumn (0.18 mm ID × 20 mm, Symmetry C18, 5 μm, Waters) and separated on an analytical RP-C18 column (0.075 mm ID × 250 mm, HSS T3, 1.8 μm, Waters) using a 90 min linear gradient of 5–35% acetonitrile/0.1% formic acid (v:v) at 300 nL/min.

Qualitative LC/MS/MS analysis was performed to construct an annotated MS/MS spectral library to improve detection rates. A top25 data-dependent acquisition (DDA) method was used, with an MS survey scan of *m/z* 350–1,250 accumulated for 350 ms at a resolution of 30,000 full-width at half-maximum (FWHM). MS/MS scans of *m/z* 180–1,600 were accumulated for 100 ms at a resolution of 17,500 FWHM and a precursor isolation width of 0.7 FWHM, resulting in a total cycle time of 2.9 s. Precursors above a threshold MS intensity of 125 with charge states 2+, 3+ or 4+ were selected for MS/MS; the dynamic exclusion time was set to 30 s. MS/MS activation was achieved by CID using nitrogen as a collision gas and the manufacturer’s default rolling collision energy settings. Fractionated rich media samples were analyzed in technical duplicate, and membrane and soluble biological samples in technical triplicate by repeated injections.

For quantitative analysis, LC/MS/MS data were acquired by data-independent acquisition (DIA) using 65 variable size windows (Zhang et al., 2015) across the 400–1,050 *m/z* range. Fragments were produced using rolling collision energy settings for charge state 2+, and fragments acquired over an *m/z* range of 350–1,400 for 40 ms per segment. In combination with a 100 ms survey scan, the overall cycle time was 2.75 s. Each biological replicate was analyzed by triplicate injection.

### 2.4 Mass spectrometry data processing

Mass spectrometry data were processed using Spectronaut v.14.2 software (Biognosys) (Bruderer et al., 2015). A hybrid spectral library was generated using the Pulsar search engine by searching all DDA and DIA data against an in-house *Hfx. volcanii* protein sequence database (version 06/2019) containing 4,107 protein entries and 79 additional noncoding ORFs^1^ augmented with 52 common lab contaminants. Protein identification was performed using default settings (enzyme specificity Trypsin/P, 2 missed cleavages; fixed Iodoacetamide (C), variable Oxidation (M), Acetylation (N-term); dynamic mass and retention time recalibration) to an estimated False Discovery Rate (FDR) of 1%, resulting in a spectral library containing 46,172 precursors mapping to 27,039 primary peptide sequences.

For protein quantitation, the spectral library was used to extract fragment ion chromatograms from the DIA data (dynamic mass and retention time alignment, up to 6 fragments per peptide, up to 10 peptides per protein, 1% FDR) resulting in quantitative values for 29,333 precursors mapping to 20,374 primary peptide sequences and 2,262 protein groups (2,254 *Hfx. volcanii*, 8 laboratory background). Quantitative data were normalized using quantile normalization, resulting in median coefficients of variation within each biological state below 20.1 and 30.0% on the peptide and protein levels, respectively.

### 2.5 Data analysis and functional annotation

Protein quantitation results were analyzed in Perseus v1.5.6.0 (Max Planck Institute of Biochemistry) (Tyanova and Cox, 2018). After grouping of individual data files for biological states and logarithmic transformation, missing values were imputed from a low-level normal distribution (width 0.3, downshift 1.8) and abundance changes analyzed for statistical significance by pairwise Student’s *t*-testing with an FDR-based multiple testing correction (*p* < 0.05, S0 = 1). Differentially abundant protein populations were analyzed for enrichment of arCOGs (Makarova et al., 2007, 2015) using FunRich v3.1.3 software (Pathan et al., 2015), and for enrichment of KEGG classes (Kanehisa and Goto, 2000) using DAVID Functional Annotation Tool v6.8 (Huang da et al., 2009).

### 2.6 Generation and analysis of deletion strain ΔHVO_A0467

For the deletion of HVO_A0467, plasmid pTA131-up.do (HVO_A0467) (Supplementary Table S1) was generated by cloning a fragment of 467 bp upstream (including the first base pair of HVO_A0467 since this is also part of the upstream gene HVO_A0466), and a fragment of 426 bp downstream of HVO_A0467 into the pTA131 vector. Subsequently strain H119 was transformed with this plasmid and plated on plates lacking uracil to mediate integration of the plasmid into the genome. Pop-in candidates were validated by PCR and streaked out on agar plates containing 5-fluoroorotic acid to force pop-out of the plasmid backbone. Pop-out candidates were investigated further by Southern blot analysis using 10 μg of genomic DNA digested with SalI and separated on a 0.8% agarose gel. DNA fragments were transferred to a nylon membrane Hybond-N+ (GE Healthcare) by capillary blotting. For verification of the knockout, two probes were used that bind either to the gene or next to the deleted region. A 362 bp fragment of the region upstream of HVO_A0467 was amplified using primers HVO_A0467-up-fwd and HVO_A0467-up-rev. A 255 bp fragment of the gene itself was amplified using oligonucleotides HVO_A0467-intern-fwd and HVO_A0467-intern-rev to serve as a gene-specific probe. The PCR fragments were labeled with [α-^32^P]-dCTP using the DECAprime II DNA labeling kit (Thermo Fisher Scientific). The labeled products were used as hybridization probes. The resulting deletion strain was termed HV118 (Supplementary Figure S1). The deletion strain was compared to wild type cells using light microscopy and growth analysis. For phase-contrast-microscopy, cells were grown in YPC or adjusted media preparations (see “Strains and Growth conditions”) until they reached the desired OD_650_ indicating the culture to be in the exponential or stationary growth phase. The OD_650_ for exponential and stationary phase differed depending on the medium used: exponential phase: YPC: OD_650_: 0.4–0.6; Hv-min: OD_650_: 0.2–0.4; Hv-min Low iron: OD_650_: 0.1–0.3; stationary phase: YPC: OD_650_ > 0.9; Hv-min: OD_650_ > 0.5; Hv-min Low iron: OD_650_ > 0.3. A 2 μL sample of culture was placed on an agarose pad prepared by dropping ~50 μL of 1% agarose containing 18% BSW (Dyall-Smith, 2008) onto a glass slide at room temperature, and a clean 22 × 50 mm number 1.5 glass coverslip placed on top. Images were acquired using a 100 × / 1.4 NA oil immersion objective and phase contrast optics using a Leica DM6 B Microscope (Leica Biosystems).

For growth experiments strains H119 (wild type) and ∆HVO_A0467 were precultured in YPC to OD_650_ = 0.4–0.6, washed once with the adjusted medium (see above “Strains and growth conditions”) and then diluted to OD_650_ = 0.05 and transferred to microtiter plates in biological triplicates and 5 technical replicates. These were subsequently cultured in a heated plate reader (Epoch 2 NS Microplate Spectrophotometer, Agilent Technologies) (aerobically, orbital shaking at 425 rpm, 45°C) while OD_650_ was measured every 30 min. Outer wells were filled with salt water as evaporation barriers (Jaschinski et al., 2014).

### 2.7 SDS-page

Cells were grown in Hv-min with or without the addition of FeSO_4_ to an OD_650_ of 0.4–0.6 before they were harvested at 10,000 × g at 4°C for 30 min. Cell pellets were washed in enriched PBS and then lysed by resuspending in lysis buffer and ultrasonication. After centrifugation (30 min, 18,000 × g, 4°C) the protein concentration of the supernatant fraction was determined using a Roti-Nanoquant assay according to the manufacturer’s protocol. 5 μg were loaded onto a 10% polyacrylamide gel. Subsequently the gel was stained with Coomassie Brilliant blue.

### 2.8 Ligand docking

The AlphaFold predicted 3D structure of *Hfx. volcanii* protein HVO_A0467 was downloaded as a pdb file (AF-D4GRD2-F1-model_v3.pdb, accessed 24/5/2023) from UniProtKB entry D4GRD2.^2^ The protein was inspected using PyMOL (v.2.4.0, Schrödinger) and checked to ensure there were no extraneous molecules. The schizokinen structure was downloaded from the Zinc15 database^3^ in SMILES format. The CB-Dock2 server (cadd.labshare.cn/cb-dock2) was used for protein-ligand blind docking (Liu et al., 2022). The SMILES structure of schizokinen was input into CB-Dock2 using the ‘draw ligand’ option. CB-Dock2 automates the preparation of ligand and protein for docking, determines potential binding pockets of the protein, and uses AutoDock Vina for ligand docking (Eberhardt et al., 2021). The docked ligand was examined further using Pymol and PLIP (Adasme et al., 2021).

### 2.9 Analysis of HVO_A0467 and HVO_A0466 homologs by BLASTp and phylogenetic tree reconstructions

Twenty one species with a completely sequenced genome were selected that represent 18 genera from 3 orders of *Halobacteriaceae* (Supplementary Table S2). First, RND permeases were selected based on their annotation from 11 species which are under continuous annotation review (category A) (Pfeiffer and Oesterhelt, 2015). BLASTp analysis confirmed that this collection is exhaustive. Similarly, all HVO_A0466 homologs were selected (annotated as RND-T-associated protein). BLASTp analysis identified two additional solo homologs (annotated as uncharacterized). From the genomes of category B, all proteins having the term RND in the protein name were also selected. The complete set of HVO_A0467 homologs was subjected to BLASTp analysis against all represented species in order to identify additional homologs. BLASTp matches which are attributed to extensive coiled-coil regions were ignored. Similarly, the complete set of already identified HVO_A0466 homologs was subjected to BLASTp analysis against all represented species in order to identify additional homologs.

For phylogenetic tree reconstructions, the webservice at https://mafft.cbrc.jp/alignment/server/ was used. Sequences in fasta format were uploaded, aligned with the MAFFT aligner (default settings) and trees inferred using the Neighbor-Joining algorithm (JTT model, 100 bootstrap sampling), and viewed using the archaeopteryx.js tool (Kuraku et al., 2013).

### 2.10 Additional bioinformatic analysis

Protter software^4^ (Omasits et al., 2014) was used to illustrate the transmembrane topology of HVO_A0467. For HVO_A0467, transmembrane domains were loaded from UniProtKB entry D4GRD2. The localization of the N-terminus was set manually to cytoplasmic, so that the large extramembrane domains are periplasmic. This is consistent with other RND permeases with available 3D structure (PDB:6AJJ; PDB:5KHS). This is also consistent with the topology prediction of TMHMM^5^ (Krogh et al., 2001) and DeepTMHMM^6^ (Hallgren et al., 2022), but opposite to the topology prediction by Phobius^7^ (Kall et al., 2007).

## 3 Results

### 3.1 Biological characterization of *Haloferax volcanii* under iron starvation conditions

**Figure 1:**
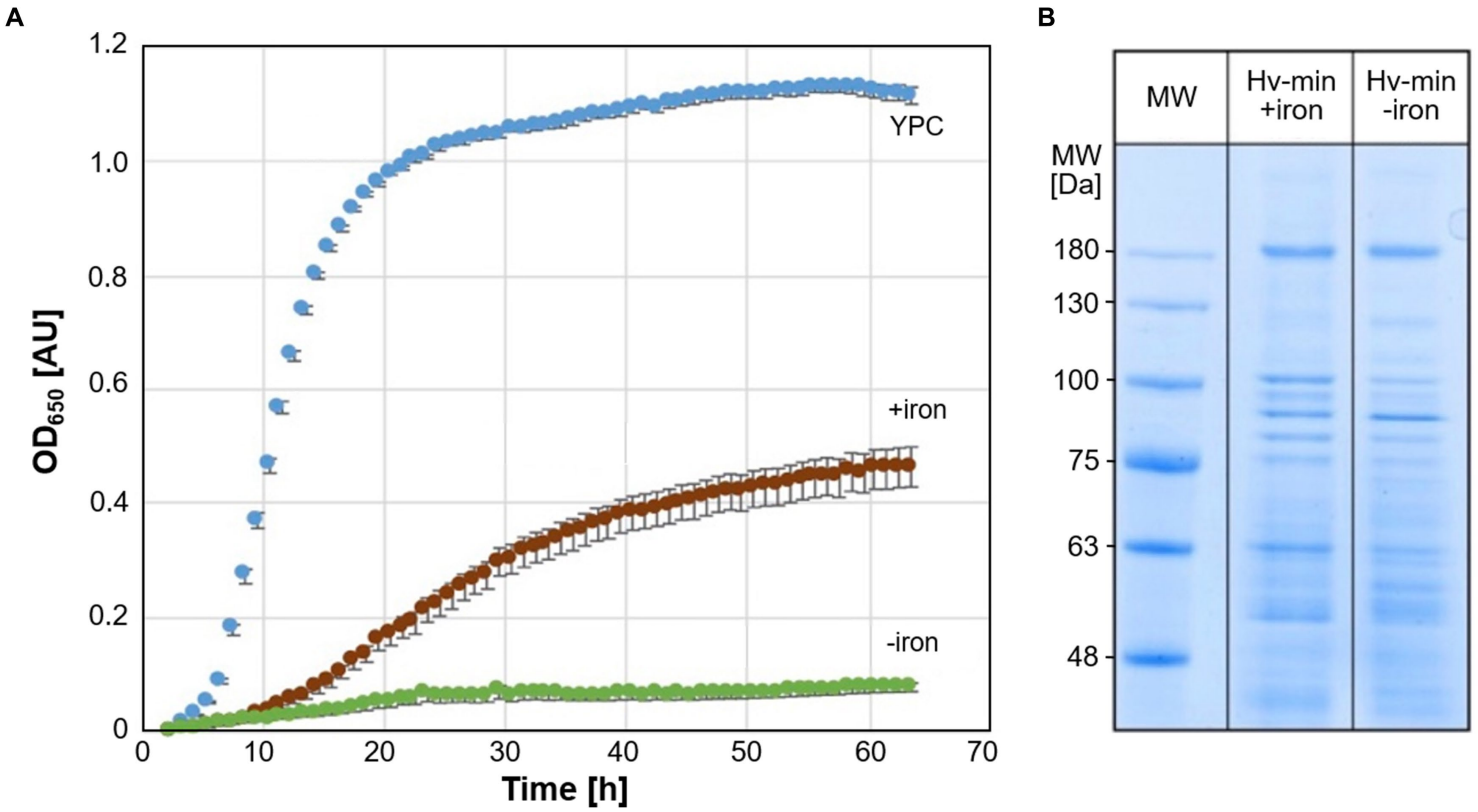
Growth of *Hfx. volcan*ii and SDS-PAGE analysis of cell proteins. **(A)** Growth of *Hfx. volcanii* H119 in minimal medium (Hv-min) with addition of FeSO_4_ (brown, +iron) or without added iron (green, −iron), and for comparison in complex medium (blue, YPC) at 45°C. *n* = 3 biological replicates, with 5 technical replicates each. y-axis: optical density at 650 nm, x-axis: time of growth in hours. **(B)** SDS-PAGE analysis of *Hfx. volcanii* H119 grown in minimal medium with (+iron) or without (−iron) addition of FeSO_4_. MW: protein apparent molecular weight marker.

Growth of *Hfx. volcanii* strain H119 was analyzed in minimal medium under conditions of iron starvation and at normal iron levels, and for comparison in complex medium (Figure 1A). While growth in minimal medium was reduced compared to complex medium, the growth of cells under iron starvation was severely attenuated. SDS-PAGE of cell extracts obtained from cultures grown with and without addition of FeSO_4_ exhibited only moderate differences in protein patterns (Figure 1B).

**Figure 2:**
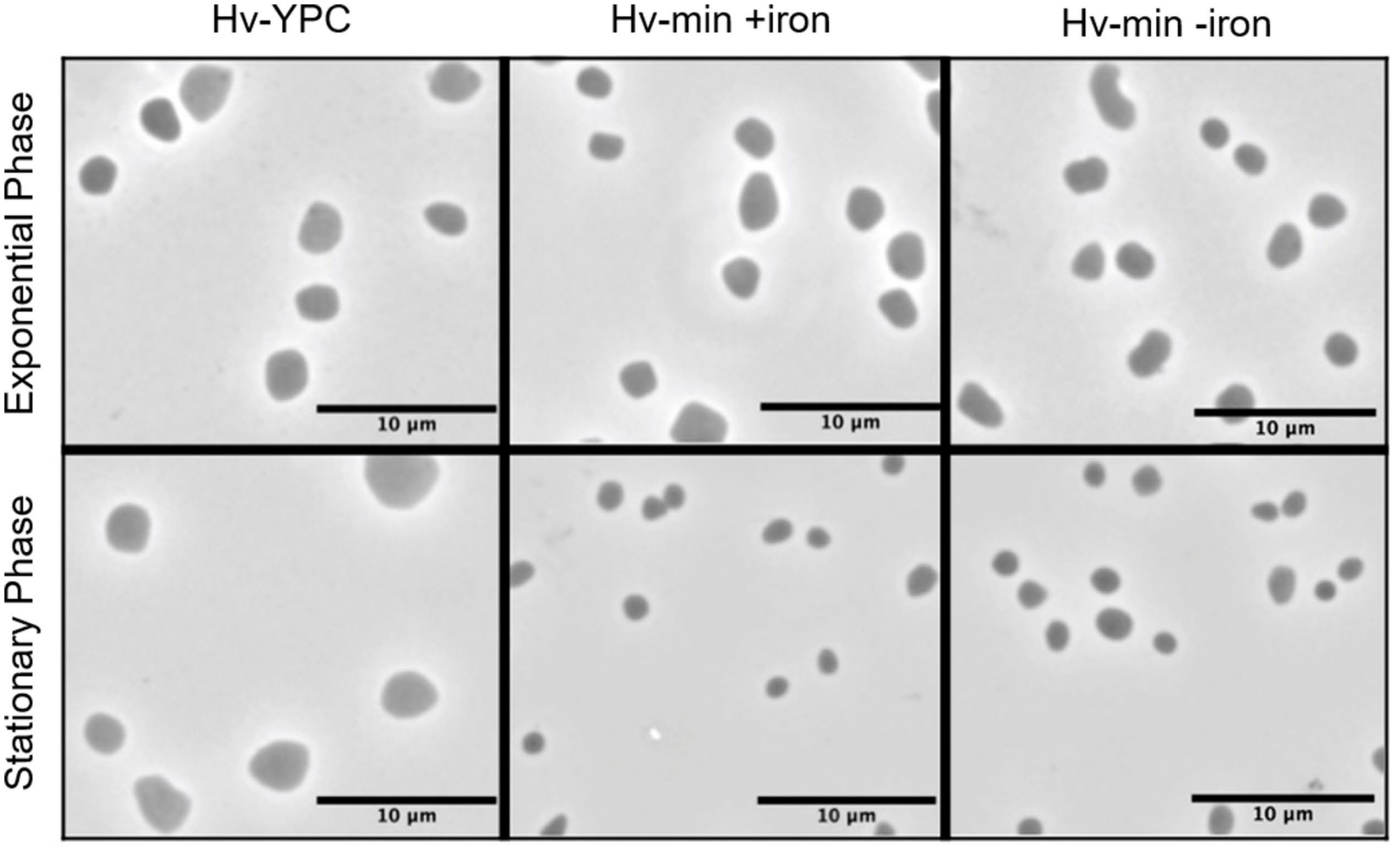
Microscopic analysis of cell morphology in the presence and absence of FeSO_4_. Phase contrast images of *Hfx. volcanii* H119 cells grown in complex medium (YPC, left panel), in minimal medium (Hv-min) with (+iron, middle panel) or without added FeSO_4_ (−iron, right panel), to exponential (upper panels) or stationary phase (lower panels). The size bar represents 10 μm.

Cells are generally smaller when grown in minimal medium instead of complex medium, but iron starvation did not cause any discernible differences in cell morphology (Figure 2). Cells were observed in late exponential and stationary phase; analysis at early exponential phase was not performed due to severe growth attenuation in the absence of iron.

### 3.2 Large-scale proteome analysis of *Haloferax volcanii* under iron starvation conditions

*Hfx. volcanii* cells grown in minimal medium with or without added iron were separated into soluble and membrane fractions, and their respective proteomes analyzed. As a first step, comprehensive protein identification was performed using both data-dependent acquisition (DDA) and data-independent acquisition (DIA) data of the fractionated samples, as well as of a reference culture grown in complex medium. We added the latter to maximize proteome coverage, as some proteins may not have been highly expressed upon growth in minimal media, and thus would not have been represented in the annotated MS/MS spectral library used for quantitative data extraction from the DIA data.

After mapping to UniprotKB accessions, this identified a total of 2,784 non-redundant *Haloferax* proteins (i.e., protein groups) evidenced by 27,040 proteotypic peptide sequences at a False Discovery Rate (FDR) of 1%. This corresponds to a coverage of 68.3% of the theoretical proteome, and compares favorably with the numbers reported by the community Archaeal Proteome Project (ArcPP) (Schulze et al., 2020). This data set includes the first mass spectrometric evidence for 12 protein products from the *Hfx. volcanii* protein sequence database which are not contained in the ArcPP data set. The identified peptide and protein identifications were transcribed into an annotated MS/MS spectral library.

**Figure 3:**
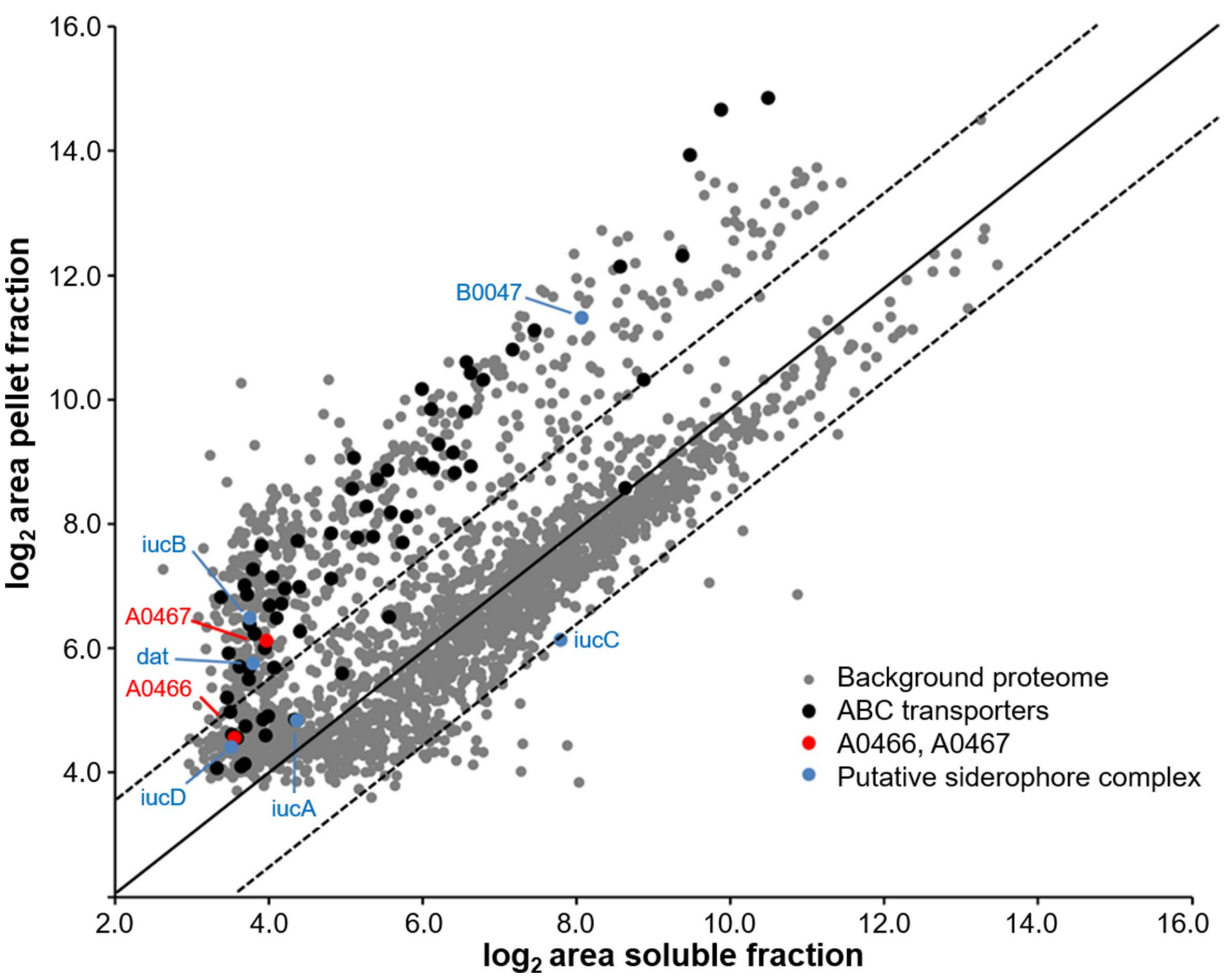
Enrichment of membrane-associated proteins in the pellet fraction. Observed log_2_ protein areas in the soluble and pellet fractions. Black, ABC type transport proteins (dppX/tsgX/znuX genes); red, proteins HVO_A0466 and HVO_A0467 investigated in this study; blue, putative siderophore complex proteins (Niessen and Soppa, 2020). Black line indicates equal abundance, dashed lines a 3-fold enrichment or depletion in the membrane fraction.

Next, we performed DIA-MS-based quantitative profiling of the membrane and soluble proteomes of *Hfx. volcanii* under iron starvation as well as under normal iron condition. Using the above spectral library, we obtained quantitative data for a total of 2,254 *Haloferax* proteins (1,786 ± 147 per injection), spanning 5 orders of magnitude linear abundance range as estimated by their iBAQ values (Schwanhausser et al., 2011) and corresponding to 55.4% of the theoretical proteome (Supplementary Table S3). To assess any methodological bias in the detected proteins, a comparison of the functional classes of this set to those of the entire proteome was conducted (see Supplementary Text S1; Supplementary Figure S1). No significant bias was detected. We also tested for the enrichment of membrane-associated proteins in the membrane fraction, and found a systematic enrichment of, e.g., ABC-type transport system proteins corresponding to the *dppx*/*tsgX*/*znuX* genes as *bona fide* membrane or membrane-associated proteins in the pellet fraction to validate our experimental approach (Figure 3).

### 3.3 Differential protein expression under iron starvation conditions

**Figure 4:**
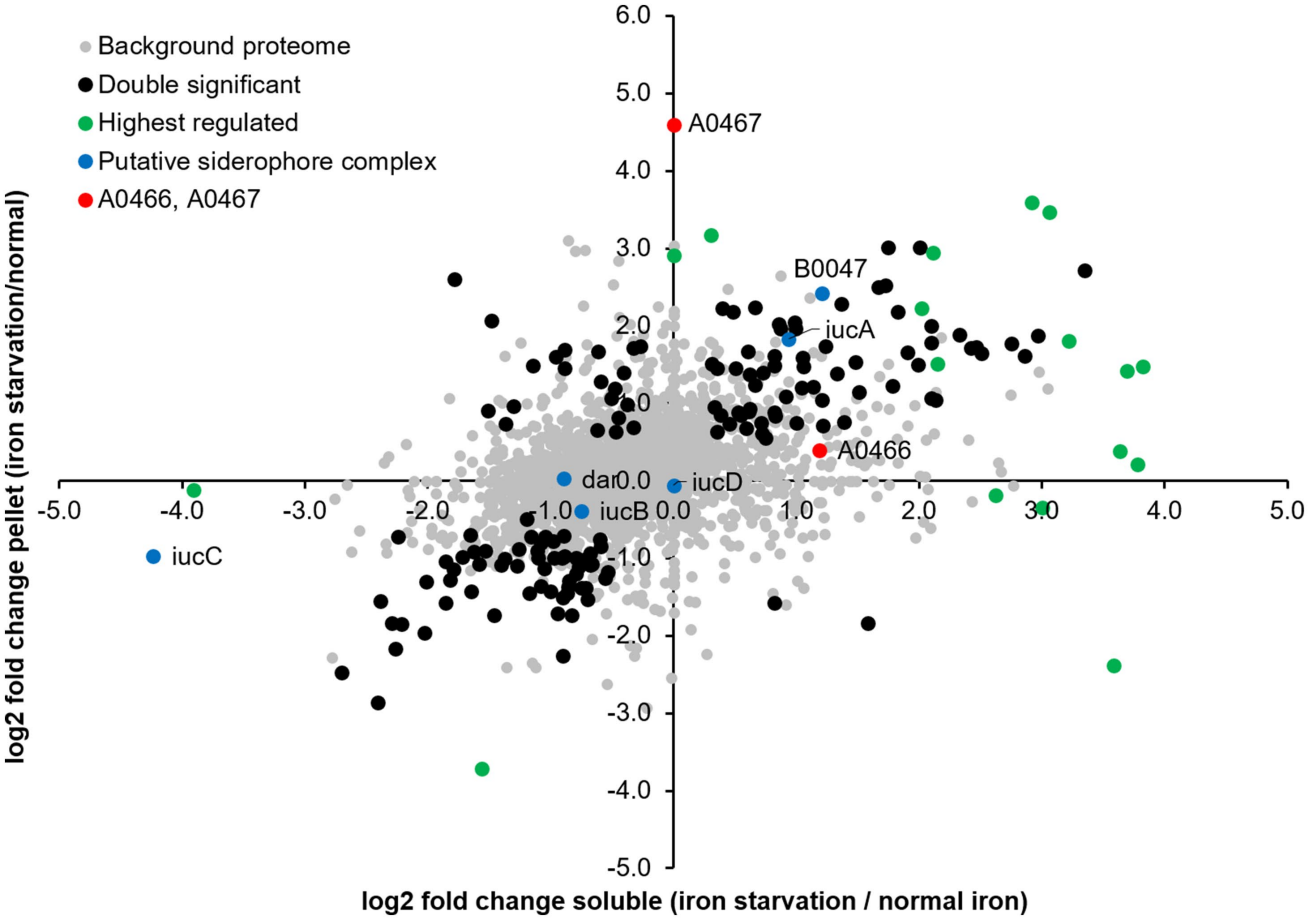
Differential protein expression under iron starvation condition. log_2_ fold changes observed in the soluble and membrane fractions relative to normal iron conditions. Black, proteins significantly differentially expressed in both soluble and pellet fractions; red, proteins HVO_A0466 and HVO_A0467 investigated in this study; green, other highest regulated proteins; blue, putative siderophore complex proteins (Niessen and Soppa, 2020).

We examined abundance changes in the membrane and soluble fractions caused by iron starvation for statistical significance by pairwise testing (Supplementary Table S4; Figure 4), and observed a total of 1,429 differentially expressed proteins: 1,138 exclusively in the soluble fraction, 136 exclusively in the pelleted membranes, and 155 in both fractions. The large number of differently expressed proteins indicates a significant fine-tuning of the *Hfx. volcanii* proteome under iron starvation conditions.

To find commonalities in the complex data, we subjected the populations of differentially abundant proteins to functional enrichment analysis, both with regard to their associated arCOG classes and to their KEGG metabolic circuits (see Supplementary Text S2). ABC transporters are among the most prominent class of proteins which were found differentially regulated. Interestingly, we do not see major changes in proteins related to stress response.

The most highly regulated proteins in our data set in either the membrane or soluble fraction were examined in detail. In total, 118 proteins were highly regulated (≥4-fold abundance change), with 79 proteins being highly up-regulated and 39 proteins being highly down-regulated (Supplementary Table S5). Table 1 lists the 19 proteins with at least 10-fold abundance changes.

**Table 1:** Proteins that are most affected by iron starvation.

Gene	Fold regulation	Protein name
**Proteins more than 10 fold up-regulated under iron starvation condition**		
HVO_A0467	+43.7	RND superfamily permease
HVO_2175	+17.7	Smc-like protein Sph3
HVO_1017	+15.6	Ketosamine kinase domain protein
HVO_1958	+14.5	Pyruvoyl-dependent arginine decarboxylase
HVO_0834	+14.4	DUF35 family protein
HVO_1004	+14.1	Conserved hypothetical protein
HVO_2009	+14.0	Conserved hypothetical protein
HVO_A0090	+13.4	Gentisate 1,2-dioxygenase
HVO_A0549	+13.0	NosL family protein
HVO_0655	+12.7	GalE family epimerase/dehydratase
HVO_B0197	+12.7	ABC-type transport system permease protein (probable substrate siderophore)
HVO_2354	+12.5	Conserved hypothetical protein
HVO_1422	+12.3	XerC/D-like integrase
HVO_2795	+12.0	GNAT family acetyltransferase
HVO_1859	+11.6	UPF0104 family protein
HVO_2359	+11.6	Conserved hypothetical protein
**Proteins more than 10 fold down-regulated under iron starvation**		
HVO_A0584	−13.8	Inosine-5′-monophosphate dehydrogenase
HVO_1676	−12.5	Transcription initiation factor TFB
HVO_B0041	−10.4	Siderophore biosynthesis protein IucC

Data for the soluble and membrane fraction are combined, and the higher of the two regulation factors is given for proteins which were found significantly regulated in both samples. Upper set, proteins more than 10 fold up-regulated under iron starvation condition. Lower set, proteins more than 10 fold down-regulated under iron starvation.

HVO_A0467 is the most highly induced protein, having an outstanding up-regulation (44-fold) specifically in the membrane fraction. It is an integral membrane protein, distantly related to the superfamily of RND family permeases, and further evidence provided below (next section) indicate it is a likely siderophore exporter. The second-most highly up-regulated protein is the SMC-like protein Sph3, which was recently shown to be required for rod formation (Schiller et al., 2024). However, cell morphology analysis was not performed in early exponential phase (where cells would form rods in the presence of Sph3) because of the limited growth at low iron conditions, with OD_650_ typically well below 0.1. Among the remaining 19 proteins showing at least 10-fold up-regulation is an ABC transporter permease subunit (HVO_B0197, fhuG2). The *fhuCGD* ABC transporters have an iron-loaded siderophore as a probable substrate according to regulon-based substrate assignment, and all *fhu* genes/operons are preceded by a *ferR* regulon signal (Leyn and Rodionov, 2015) (see Supplementary Text S5). HVO_B0197 is one of two paralogous permeases and shows 12.7-fold up-regulation. There are 11 paralogous substrate-binding proteins of the iron-loaded siderophore ABC transporter (*fhuD*), 5 of which are up-regulated more than 4-fold (HVO_1464, 8.0-fold; HVO_A0557, 7.0-fold, HVO_B0047, 5.2-fold, HVO_B0144, 4.9-fold and HVO_B0198, 4.5-fold). None of the remaining, highly up-regulated proteins with known function appear to show a direct relation to iron transport and metabolism. Many of the regulated proteins lack a definite function assignment or are totally uncharacterized, i.e., are annotated as conserved hypothetical protein.

The most highly down-regulated protein (HVO_A0584, 13.8-fold) has no obvious connection to iron starvation. Down-regulation of a general transcription factor (HVO_1676, *tfb2*, 12.5-fold) may indicate multi-layer gene regulation, as general transcription factors have been implicated in gene regulation in haloarchaea (Coker and DasSarma, 2007; Lu et al., 2008). Other transcription factors are also down-regulated (HVO_2268, *tfb4*, 5.9-fold, HVO_1052, *tfb1*, 4.7-fold). The down-regulation of HVO_B0041 (*iucC*, 10.4-fold) is counter-intuitive for a siderophore biosynthesis protein. Another protein from the same operon, HVO_B0044 (*iucA*) is oppositely regulated (3.8-fold up-regulation). More details about the siderophore biosynthesis operon are given in Supplementary Text S6. Finally, one subunit of a respiratory complex I homolog (Nuo complex) is strongly down-regulated (NuoN, 9.9-fold). Of the 11 subunits of the Nuo complex, 9 were found to be down-regulated. Also, additional subunits of the respiratory chain were found to be down-regulated (Supplementary Text S4).

Besides looking at the highest differentially abundant protein, we also examined the expression of known iron-containing proteins. Indeed we consistently found slightly (~2fold), but statistically significantly reduced expression of a number of cytochrome complex proteins (e.g., HVO_0038 *cyc1*, HVO_0841 *petD*, HVO_0842 *petB*), ferredoxins (HVO_2995 *fdx*, HVO_1831 *ferA4*, HVO_2729 *ferB2*) and ferredoxin-related proteins (HVO_0869 *gltB*, HVO_0887 *korB*, HVO_0888 *korA*, HVO_1304 *porB*, HVO_1305 *porA*) as well as other putative iron–sulfur proteins (HVO_A0083, HVO_1695) in the cytosol under iron starvation conditions. This reduced expression of key components of the redox chain reflects the effects of iron starvation on cellular metabolism, and highlights the importance of elucidating potential siderophore systems in more detail.

### 3.4 HVO_A0467, a potential siderophore exporter, is the most highly regulated protein

**Figure 5:**
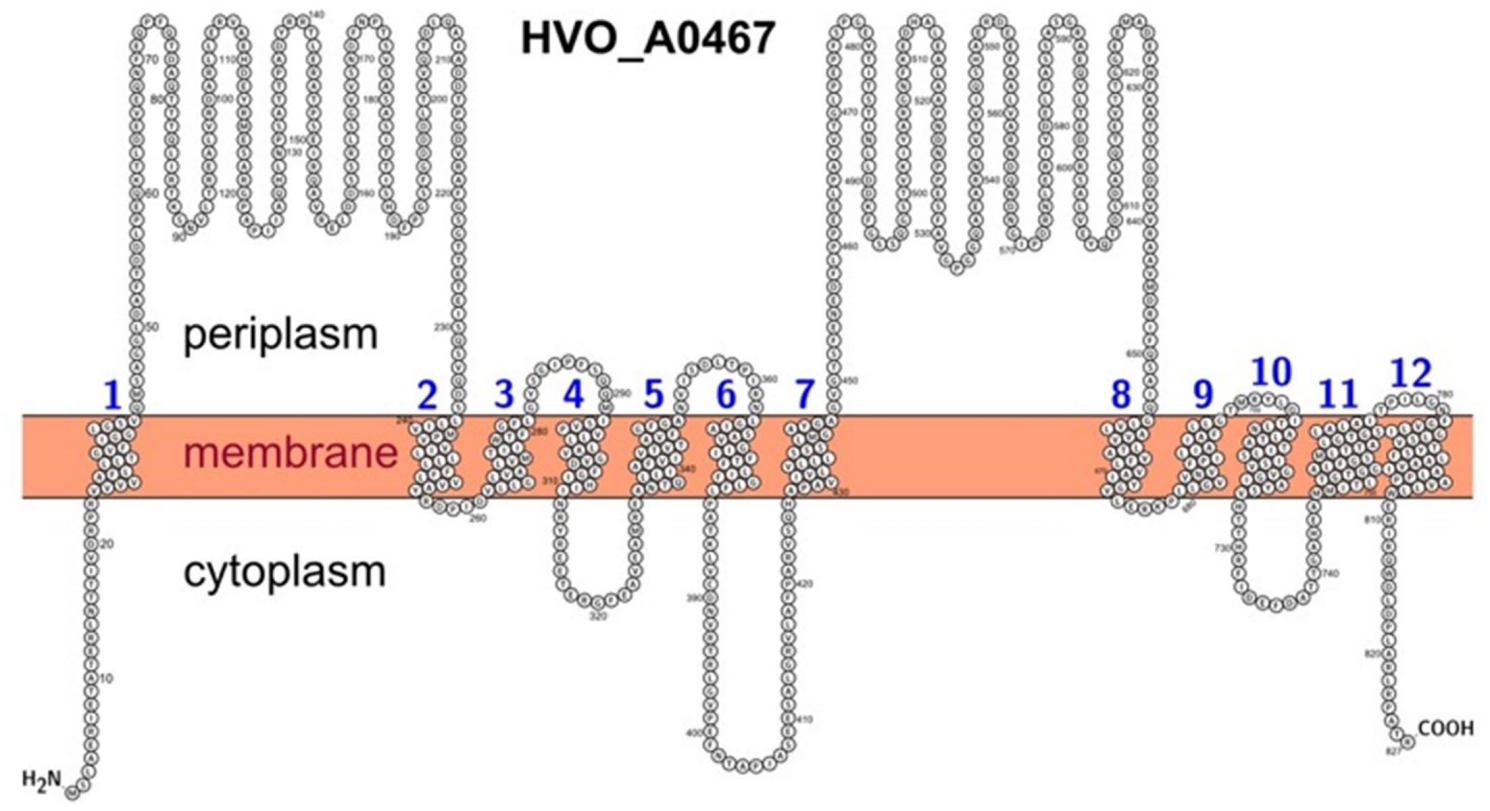
Predicted membrane topology of HVO_0467 as depicted using the PROTTER webserver. The transmembrane domains are based on UniProt features and are numbered from 1 to 12. The N-terminus was set to intracellular as predicted by DeepTMHMM and TMHMM, contrary to a Phobius prediction.

As pointed out above, HVO_A0467 is the most highly regulated protein, being up-regulated 44-fold under iron starvation conditions (Figure 4; Table 1). It is an 827 amino acid integral membrane protein with 12 predicted transmembrane domains (Figure 5) but was undetected in many previous proteome studies, such as the 12 MS datasets used for the original Archaeal Proteome Project (arcPP) (Schulze et al., 2020). A later dataset (Schulze et al., 2021) detected two peptides, however each only with a single peptide-to-spectrum match. This is in line with its observation at very low levels in our data under normal iron concentrations. In our proteomic data set it is reliably detected with 3 peptides (^387^LVCDNVRTR^395^, ^578^IYDELFASSASGR^590^, ^632^ATSTGDVVVR^641^).

To gain further insight into the function of this protein, a bioinformatic approach was used to detect relationships with distant homologs. HVO_A0467 is an RND superfamily transport protein, based on BLASTp comparison to the transporter classification database (TCDB) (Saier et al., 2021). The top hits are transporters (permeases) of the RND (Resistance/Nodulation/Cell division) superfamily. There are only extremely distant homologs in the annotated SwissProt section of UniProtKB, with the best hit (26% sequence identity) being uncharacterized. The N-terminal and C-terminal halves of RND transporters share sequence similarities, each half consisting of an MMPL (Mycobacterial Membrane Protein Large) domain (InterPro: IPR004869; Domenech et al., 2005). *Mycobacterium* codes for 12 RND transporters, one of which was shown to be involved in siderophore export (Wells et al., 2013).

**Figure 6:**
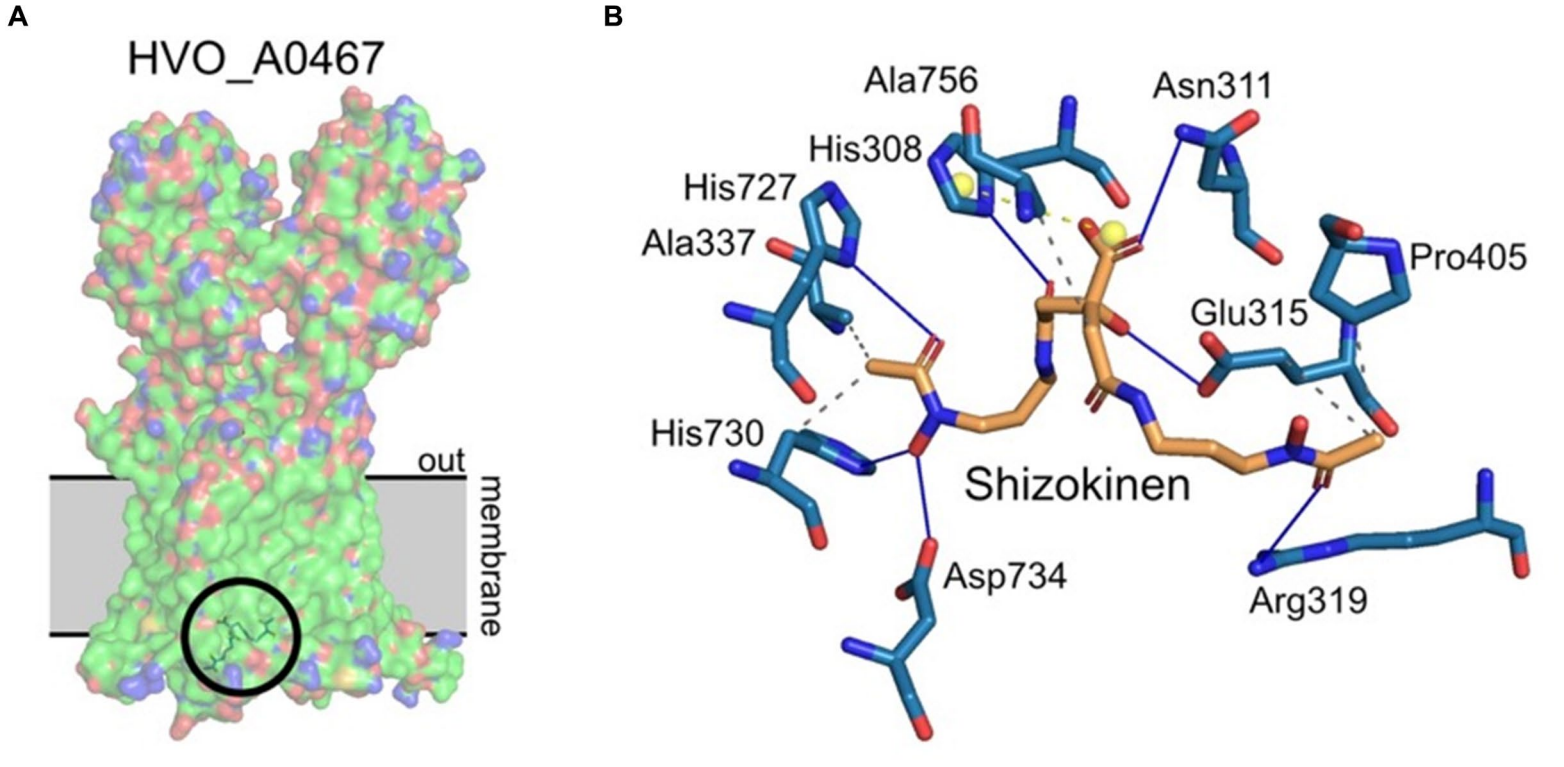
Protein/ligand docking of schizokinen to HVO_A0467. **(A)** Lowest energy binding pose of schizokinen (circled) to HVO_A0467, as predicted by CB-Dock2. The position of the archaeal membrane (gray) was predicted using PPM3.0. The ligand lies at the base of the internal channel of HVO_A0467 formed by the 12 transmembrane domains of the protein. Surface coloring by electrostatic potential, with the hydrophobic membrane spanning trunk region largely in green. **(B)** Predicted interactions of schizokinen with the residues of HVO_A0467. Image obtained using PLIP (Adasme et al., 2021). Blue lines, hydrogen bonds; yellow balls, charge centers; gray dashed lines, hydrophobic interactions.

Protein-ligand docking was used to investigate whether HVO_A0467 might function as an exporter of the *Hfx. volcanii* siderophore schizokinen. The identity of this siderophore was proposed earlier (Niessen and Soppa, 2020) and is consistent with functional assignment by the antiSMASH server (Blin et al., 2023). Schizokinen was docked *in silico* to the 3D structure of HVO_A0467 predicted by AlphaFold using the CB-Dock2 server (Liu et al., 2022). Five potential binding pockets were detected on the surface of HVO_A0467, with the largest (C1, 3,612 Å^3^) being at the base of the transmembrane domain (TMD), a region that includes a central channel as well as residues predicted to be projecting into the cytoplasm. The C1 pocket produced the highest free binding energy for schizokinen by far (Vina score − 7.5 kcal/mol), with the other pockets located in the head domains showing lower binding energies of −5.5 kcal/mol or less. The free binding energy of −7.5 kcal/mol for the C1 pocket is similar to free binding energies of −7.7 to −8.2 kcal/mol, which have been obtained with other ligand-transporter interactions (Kumar et al., 2017; Zhang et al., 2019; Ple et al., 2022). Figure 6 shows schizokinen bound in the central channel of the C1 pocket, near the base of the TMD. It is predicted to have binding interactions with 10 sidechains.

Schizokinen is a hydroxamate-type siderophore, and is hydrophilic (Khan et al., 2018), which makes it different from the well-studied lipophilic substrates that have been used to study RND transporters, such as the mycolic acid transporter MmpL3 (Klenotic et al., 2021). Lipophilic molecules enter the cell membrane and are then taken up by RND transporters via channels that open into the membrane. Schizokinen is likely to bind initially to the cytoplasmic base of HVO_A0467, although it remains unknown if transport can be performed by a single protein, or if it needs to assemble into a homodimer or trimer.

### 3.5 HVO_A0467 is not essential under standard conditions

**Figure 7:**
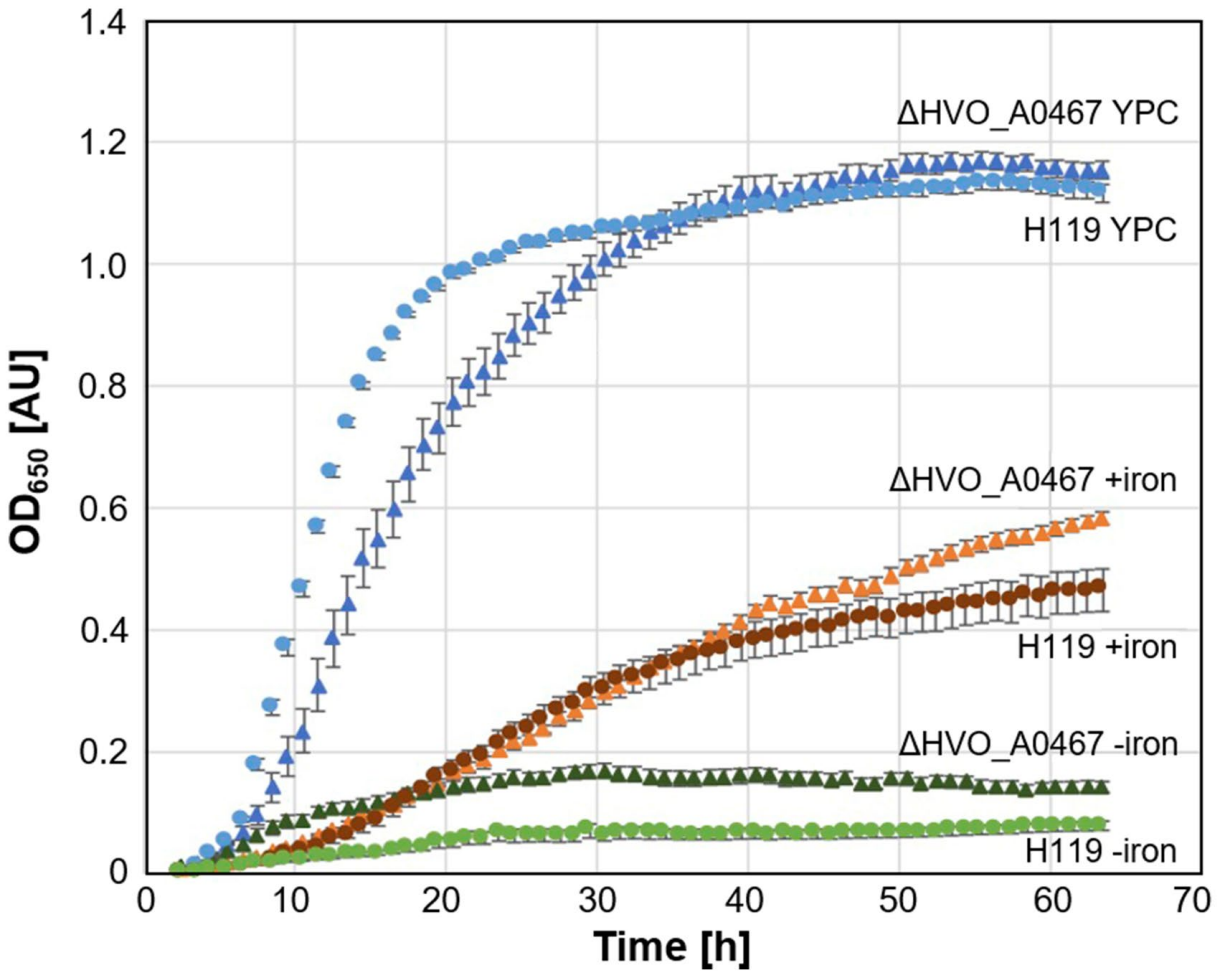
Growth of parent strain H119 and deletion strain ∆HVO_A0467 in different media. Growth of *Hfx. volcanii* H119 (circles) and ∆HVO_A0467 (triangles) in complex medium (blue, YPC), minimal medium (Hv-min) with (brown, +iron) or without added FeSO_4_ (green, −iron). *n* = 3 biological replicates, with 5 technical replicates each. y-axis: optical density at 650 nm, x-axis: time of growth in hours.

Deletion of the HVO_A0467 gene produced a viable strain of *Hfx. volcanii* (ΔHVO_A0467), showing that this gene is not essential under standard growth conditions. No pronounced differences could be detected between cell shapes from the deletion strain and its parent under different growth conditions (data not shown). Interestingly, the deletion mutant grows slightly better in minimal medium and low iron (Figure 7).

### 3.6 HVO_A0467 forms an operon with HVO_0466

**Figure 8:**
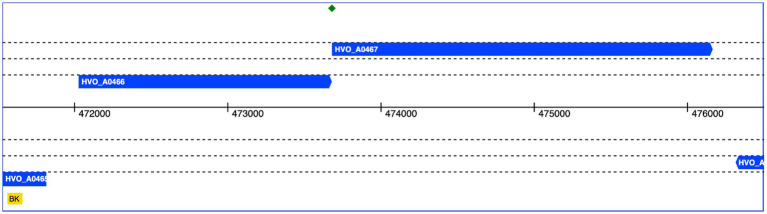
Genomic location of HVO_A0466 and HVO_A0467. Both genes overlap by one nucleotide (green diamond). The genomic coordinates of pHV4 are shown in the middle. Data obtained from HaloLex (Pfeiffer et al., 2008).

The genomic context of HVO_A0467 was found to be intriguing. HVO_A0467 forms an operon with HVO_A0466 (Figure 8), which encodes an uncharacterized protein that was shown to be glycosylated (Schulze et al., 2021) and is probably secreted. The genes have a one-base overlap, with the last base of the HVO_A0466 stop codon being the first base of the HVO_A0467 start codon.

**Figure 9:**
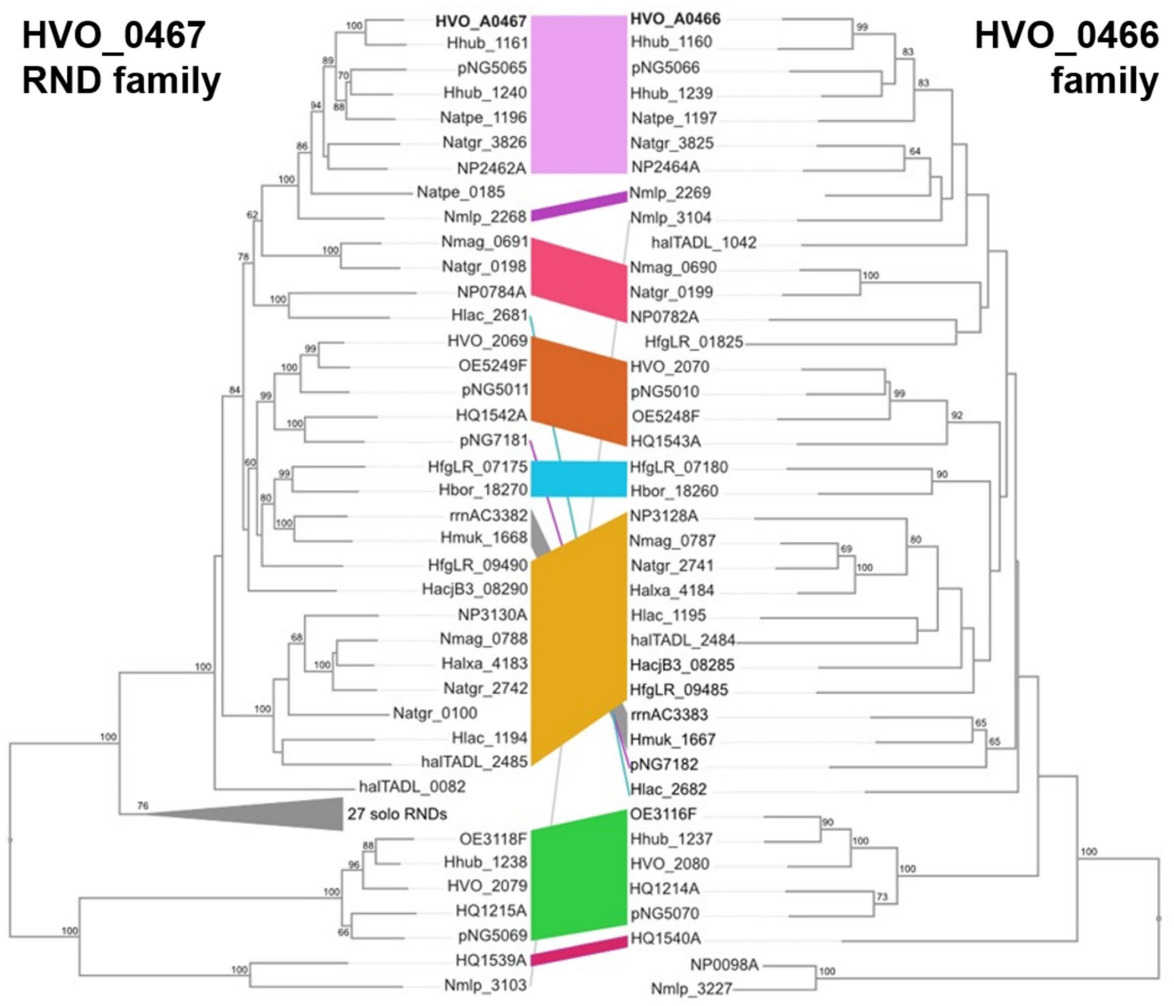
Comparison of inferred phylogenies of HVO_A0467 (RND) family proteins (left) and HVO_A0466 homologs (right). Colored stripes indicate corresponding proteins from the same genome, with many such pairs forming related groupings. For example, HVO_A0467 and HVO_A0466 (top) are bolded, and belong to a cluster of related, corresponding protein pairs (pink stripe). NJ algorithm with bootstrap values (100 replicates) of >60 shown at branch points.

An initial analysis detected two paralogs of HVO_A0466 in *Hfx. volcanii*, each of them also encoded adjacent to an RND permease. Therefore, we systematically analyzed a selection of 21 genomes from 18 genera (Figure 9; Supplementary Table S2 “Species Homologs”). With very few exceptions, HVO_A0466 homologs are encoded adjacent to an RND permease, and many RND permeases are encoded adjacent to a HVO_A0466 homolog, suggesting a shared molecular function of HVO_A0466 with HVO_A0467.

## 4 Discussion

*Hfx. volcanii* is a well-studied archaeal microorganism which has shown remarkable adaptability to changes in environmental conditions such as hypoxia, salinity or temperature stress. No information has been available, however, about its response to iron scarcity, a key environmental factor limiting microbial growth with a broad range of response mechanisms reported in bacteria. Bioinformatic analysis indicated the presence of a gene cluster for biosynthesis of the siderophore schizokinen in *Hfx. volcanii*, but solid evidence and information on the corresponding siderophore export and ferri-siderophore reimport systems has been critically lacking.

In order to deepen our understanding of the response of *Hfx. volcanii* to iron scarcity, we present the results of a differential proteome analysis conducted under conditions of iron scarcity compared to normal iron concentration. The chosen conditions lead to severe growth attenuation and thus are suitable addressing this biological phenomenon. We conducted fractionation of the samples to put increased focus on the detection of membrane proteins. Surprisingly, iron scarcity did not produce a stringent proteomics response similar to the one observed under salinity and temperature stress (Jevtic et al., 2019). More specifically, even the *iucABCD* gene cluster predicted to be responsible for schizokinen production did not show a uniform pattern of regulation, with *iucC* being strongly down-regulated.

The protein showing the highest factor of up-regulation under iron scarcity was HVO_A0467, an RND superfamily integral membrane protein with 12 predicted transmembrane proteins. Intriguingly, computational docking of schizokinen to a predicted structure of HVO_A0467 identified a potential binding pocket close to the base of the internal channel of HVO_A0467 formed by the 12 transmembrane domains of the protein, making the protein a strong candidate for export of schizokinen. Deleting HVO_A0467, however, did confer neither a growth advantage nor a disadvantage under iron scarcity, indicating that there may be alternative mechanisms of sequestering iron in *Hfx. volcanii.* Additional experimentation will be necessary to finally confirm both the production of schizokinen and its export by HVO_A0467.

The RND permease HVO_A0467 shows a strong genetic association with the upstream gene HVO_A0466 that encodes a secreted glycoprotein (Supplementary Table S6; Figure 9). This is also worth investigating, as they may interact functionally within the pseudoperiplasmic space. AlphaFold predicts HVO_A0466 to be a long, fiber-like protein consisting of four regularly spaced, closely folded domains (Ig-folds) and attached to the membrane via a C-terminal transmembrane domain (Supplementary Figure S4). The finding that such an association between RND proteins and HVO_A0466-like proteins is widespread makes it likely there is a functional interaction between RND and its cognate HVO_A0466-like protein.

A recent study of the *Hfx. volcanii* transcriptional regulatory network (TRN) identified three diphtheria toxin repressor (DtxR) proteins as potential transcriptional regulators of iron homeostasis, Idr (HVO_0538), SirR (HVO_0819) and TroR (HVO_0863) (Martinez Pastor et al., 2024). We were able to detect SirR and TroR in our proteomic data set, however neither of the two proteins showed significantly differential abundance upon iron starvation in either the soluble or the membrane fraction. This discrepancy between the transcriptome and proteome points to more complex mechanisms employed by *Hfx. volcanii* to maintain iron homeostasis under conditions of scarcity.

## Data availability statement

The mass spectrometry proteomics data have been deposited to the ProteomeXchange Consortium via the PRIDE (Perez-Riverol et al., 2019) partner repository with the dataset identifier PXD029036.

## Author contributions

A-LS: Data curation, Formal analysis, Investigation, Methodology, Visualization, Writing – original draft, Writing – review & editing. ZJ: Data curation, Formal analysis, Investigation, Methodology, Writing – review & editing. BS: Formal analysis, Investigation, Methodology, Writing – review & editing. JW: Formal analysis, Investigation, Methodology, Writing – review & editing. KS: Formal analysis, Investigation, Writing – review & editing. HU: Conceptualization, Funding acquisition, Resources, Supervision, Validation, Writing – review & editing. MD-S: Conceptualization, Data curation, Formal analysis, Investigation, Methodology, Validation, Visualization, Writing – original draft, Writing – review & editing. FP: Conceptualization, Data curation, Formal analysis, Investigation, Methodology, Validation, Visualization, Writing – original draft, Writing – review & editing. AM: Conceptualization, Data curation, Formal analysis, Funding acquisition, Methodology, Project administration, Resources, Supervision, Validation, Writing – original draft, Writing – review & editing. CL: Conceptualization, Data curation, Formal analysis, Methodology, Project administration, Resources, Supervision, Validation, Visualization, Writing – original draft, Writing – review & editing.

## Footnotes

^1^ https://doi.org/10.5281/zenodo.3565631

^2^ www.uniprot.org/uniprotkb/D4GRD2/entry

^3^ zinc15.docking.org/substances/ZINC000014692536

^4^ https://wlab.ethz.ch/protter/start/

^5^ https://services.healthtech.dtu.dk/services/TMHMM-2.0/

^6^ https://dtu.biolib.com/DeepTMHMM

^7^ https://phobius.sbc.su.se/
