# Supplementary material for: Iron starvation results in up-regulation of a probable Haloferax volcanii siderophore transporter
Source: Front Microbiol. 2024 Aug 14;15:1422844. doi: 10.3389/fmicb.2024.1422844 (PMC11349517; doi:10.3389/fmicb.2024.1422844)
Supplement: Supplementary file 1 [file Data_Sheet_1.pdf]

Iron Starvation Results in Up-Regulation of a Probable *Haloferax volcanii* Siderophore Transporter

- Supplementary Texts and Figures -

Anna-Lena Sailer[1†], Zivojin Jevtic[2,3†], Britta Stoll[1], Julia Wörtz[1], Kundan Sharma[4], Henning Urlaub[4,5], Mike Dyall-Smith[6,7], Friedhelm Pfeiffer[1,6], Anita Marchfelder[1*], Christof Lenz[4,5*]

[1]Biology II, Ulm University, Ulm, Germany

[2]University Children's Hospital, University of Basel, Basel, Switzerland

[3]Department of Biomedicine, University of Basel, Basel, Switzerland

[4]Bioanalytical Mass Spectrometry Group, Max Planck Institute for Multidisciplinary Sciences, Göttingen, Germany

[5]Bioanalytics Groups, Department of Clinical Chemistry, University Medical Center Göttingen, Göttingen, Germany

[6]Computational Systems Biochemistry, Max Planck Institute for Biochemistry, Martinsried, Germany

[7]Veterinary Biosciences, Melbourne Veterinary School, Faculty of Science, University of Melbourne, Parkville, Australia

[†]These authors contributed equally to this work and share first authorship

[*]These authors share last authorship

*** Correspondence:**
Anita Marchfelder
anita.marchfelder@uni-ulm.de

Christof Lenz
christof.lenz@med.uni-goettingen.de

Keywords: *Haloferax volcanii*, proteome, iron starvation, data-independent acquisition mass spectrometry, DIA-MS, import/export, metal homeostasis

Supplementary Text S1: arCOG enrichment analysis of all quantified proteins

To assess a potential bias against functional classes of proteins caused by our experimental protocol, we performed arCOG enrichment analysis of the quantified proteins against the *Hfx. volcanii* proteome (Supplementary Figure S1). Indeed, we only observed ≥1.5fold over-representation of proteins assigned to nucleotide transport and metabolism (F) and protein translation (J), and under-representation of proteins involved in the Mobilome (X) and in Cell Motility (N). Since the latter arCOG classifications are somewhat problematic due to the marked differences between the bacterial and archaeal motility apparatuses, we conclude that our protocol does not exhibit significant bias against defined functional classes. The deviations are consistent with the typical high or low expression rates of proteins that belong to those functional classes. Thus, we conclude that our experimental protocol is devoid of a bias in favor or against certain subsets of proteins.

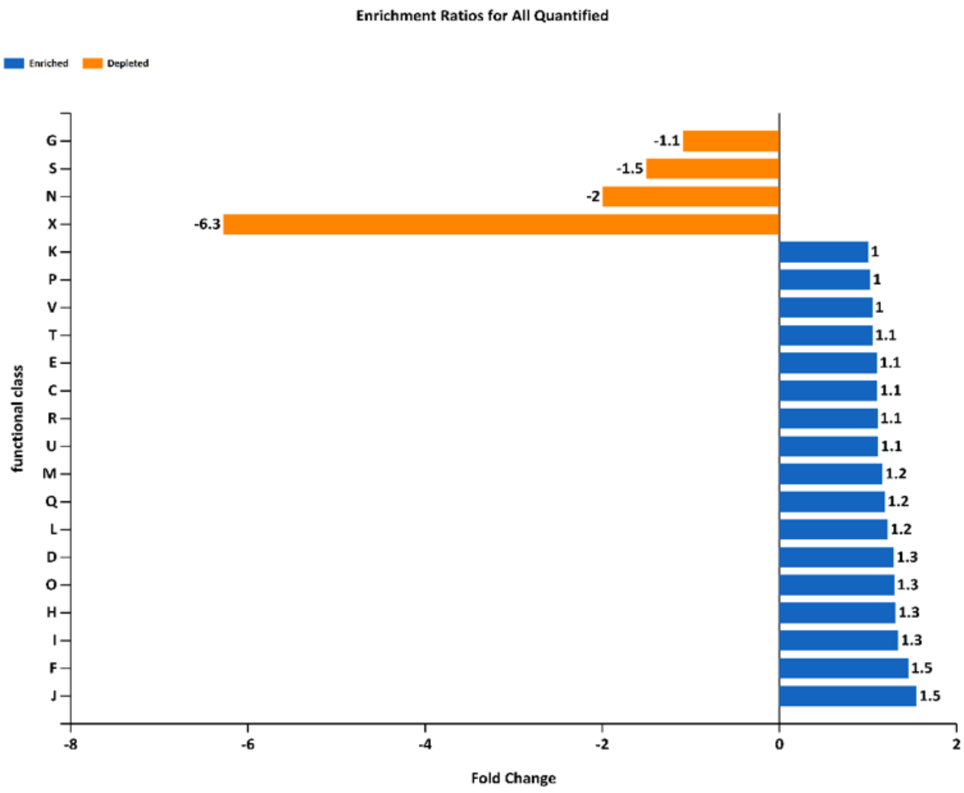

Suppl. Fig. S1. arCOG enrichment analysis of proteins quantified in our experiment. Enrichment ratios in comparison to the complete *Hfx. volcanii* proteome are displayed. For most well defined classifications, only moderate enrichment or depletion ratios were observed.

Supplementary Text S2: KEGG and arCOG enrichment analysis of differentially abundant proteins

Following differential abundant testing of the proteome data (Figure 4, Supplementary table S4), we subjected the subgroups of statistically significantly differentially abundant proteins in both the soluble and pellet fractions to functional enrichment analysis against the arCOG and KEGG classification systems. In spite of the limited biological knowledge about the function of many *Hfx. volcanii* proteins especially with regard to KEGG terms, a number of enrichments could be clearly visualized (Supplementary Figure S2).

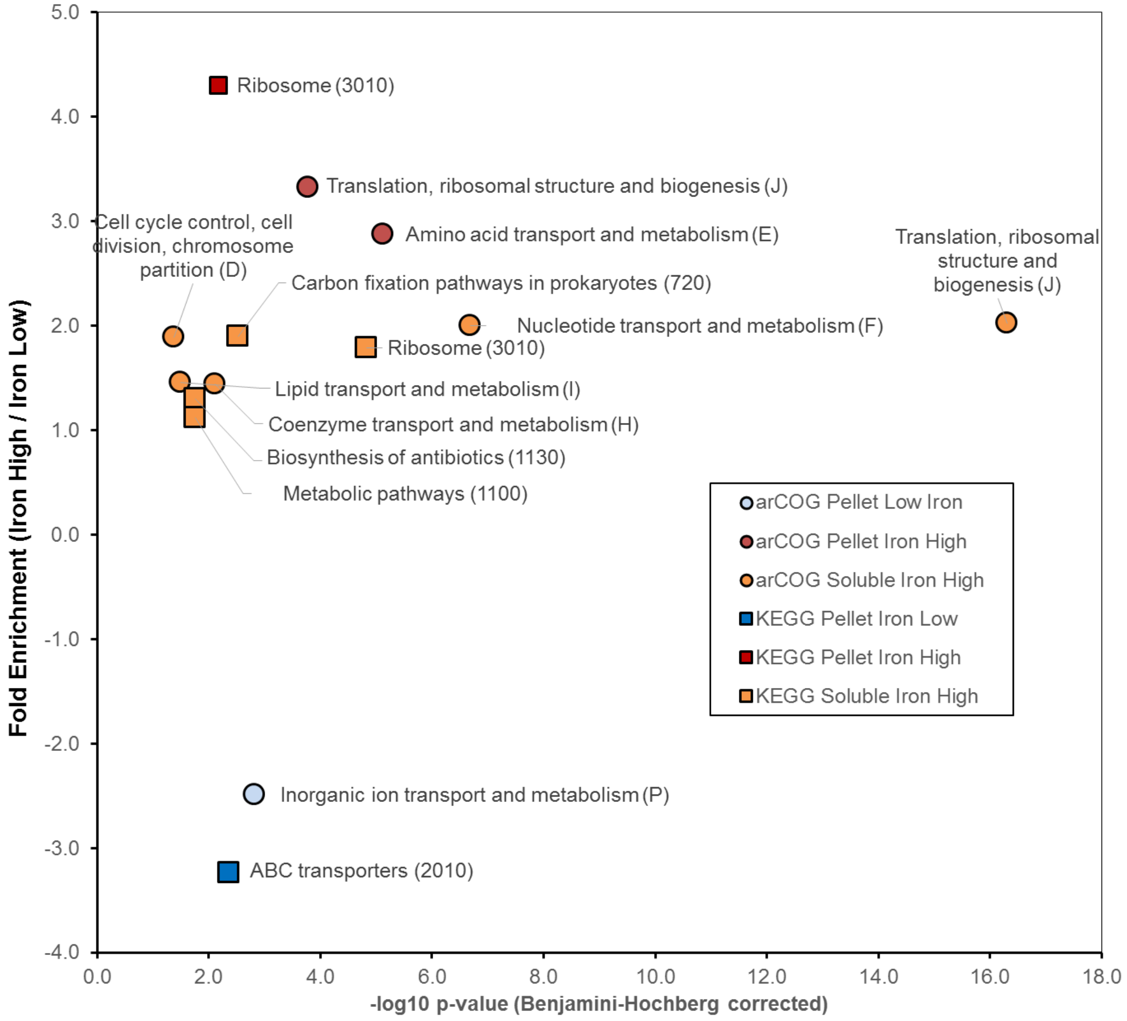

Suppl. Fig. S2. Functional enrichment analysis of differentially abundant proteins. Statistically significantly enriched arCOG classes (circles) and KEGG terms (squares) found in the pellet and soluble fractions under normal iron conditions (“Iron High”, red, orange) and iron starvation conditions (“Iron Low”, light blue, dark blue) conditions. No significant functional enrichments were found in either soluble or pellet fractions under iron starvation conditions.

Comparison of enriched arCOG classes and KEGG terms shows that iron starvation clearly stimulates the expression of transport systems found in the pellet fraction, specifically ABC transporters. Conversely, amino acid transport systems depleted under these conditions, indicating an only partially differentiated response under conditions of nutrient scarcity. Due to the often tentative

assignment of transporter specificities in archaeal databases, these differentiations should be carefully examined on a case-by-case basis. Surprisingly, and unlike the results from our earlier study on heat and salt stress in *Hfx. volcanii* (Jevtic *et al*., 2019), we did not observe significant up-regulation of other cellular processes under conditions of iron starvation. On the contrary, quite a number of cellular processes are found down-regulated under iron starvation. These include cell division, protein translation as well as a range of metabolic circuits pertaining to e.g. amino acids, nucleotides, lipids and coenzymes. On the global level this seems to reflect the overall slower growth and cell metabolism under conditions of iron starvation rather than activation of an organism-wide stress response.

Supplementary Text S3: Correlation of the soluble and membrane fractionations

A considerable fraction of the quantified proteins were detected in both the soluble and in the membrane fraction. We analyzed for the correlation of the sample with the predicted subcellular localization. We also analyzed for a consistent and discrepant direction of regulation in these samples.

Among proteins detected as regulated or highly regulated in both fractions, almost all exhibited the same direction of regulation, which probably reflects both protein partitioning between the membrane and soluble fractions as well as a limited specificity of the fractionation itself. E.g., several subunits of the respiratory chain (NuoN; NuoL; SdhD; PetB) are strongly down-regulated under iron starvation. Unexpectedly though, these and all other subunits of the respiratory chain complexes were found regulated in the soluble fraction, which might indicate that membrane pelleting by ultracentrifugation did not result in high purity. Difficulties to pellet membranes under standard centrifugation conditions have also been reported for *Hbt. salinarum* (Klein *et al*., 2005).

To shed more light on this, we next considered if the distribution of the most highly regulated proteins between soluble and membrane fraction corresponds to the anticipated cellular localization. Localization prediction data were taken from the ArcPP project (Schulze *et al*., 2020). For up-regulated proteins the correlation was acceptable: 40 out of 49 proteins (81%) in the soluble fraction were predicted to be soluble, and 18 out of 33 proteins (54%) in the membrane fraction predicted to be membrane-associated. Among the down-regulated proteins these trends were less (20 out of 34 predicted soluble proteins (59%) in the soluble fraction, 5 out of 11 predicted membrane proteins (45%) in the membrane fraction). Even so, the results of this analysis support that both the soluble and the membrane fractions overall confidently reflect the corresponding cellular environments. Even though the separation into membrane and soluble fraction only partially reflects subcellular localization, this separation may have allowed to widen the set of proteins which could be subjected to quantitative analysis as many proteins were found significantly regulated only under one of the two conditions.

Supplementary Text S4: Further biological observations in the set of proteins which are differentially expressed under iron starvation conditions

On a more fine-grained level of analysis, our data provide evidence for individual cellular processes being strongly affected by iron starvation. E.g., 9 of the 11 subunits of the Nuo complex are found down-regulated under iron starvation (NuoA,B,CD1,H,I,K,L,M,N) (2.0 to 2.4-fold for subunits other than NuoLN). The genes nuoC and nuoD are fused in haloarchaea to nuoCD, and there are two paralogs in *Hfx. volcanii*. The gene nuoCD1 is encoded within the nuo operon and is regulated consistently to the other subunits (2.1-fold down-regulated). The gene nuoCD2 is encoded separately as a monocistronic gene and is found highly and oppositely regulated (7.4-fold up-regulated in the soluble fraction). The nuoJ gene is split in haloarchaea, but neither nuoJ1 nor nuoJ2 were among the significantly regulated proteins. Overall, an astonishingly high fraction (29/79 up-regulated, 17/41 down-regulated) of the highly regulated proteins is plasmid-encoded.

Hfx. volcanii contains a siderophore biosynthesis operon (HVO_B0046-HVO_B0041) with an adjacently encoded ABC transporter binding protein (HVO_B0047). This is adressed in more detail in Suppl. Text S6 below.

Supplementary Text S5: Regulon-based substrate assignments for several haloarchaeal transporters

Hfx. volcanii has been subjected to extensive Gold Standard Protein based genome annotation (Pfeiffer and Oesterhelt, 2015). This avoids over-annotation but leaves many membrane transporters without substrate assignment. In such cases, regulon-based function prediction can be applied, such as the iron homeostasis regulon described for *Hbt. salinarum* (Schmid *et al*., 2011) and other haloarchaea (Leyn and Rodionov, 2015) . The strong conservation of DtxR transcription factor binding site motifs upstream of homologous genes in different haloarchaea allows highly convincing gene function assignments when examining related species.

Concerning the ABC-type transport system FhuCDG which is likely to transport iron-loaded siderophore, the *Hfx. volcanii* genome codes for a single ATPase (HVO_1760), two permeases (HVO_1759, HVO_B0197) and 11 paralogs of the periplasmic substrate-binding protein. Of these binding proteins, 5 (HVO_1464, 8.0-fold, HVO_A0557, 7.0-fold, HVO_B0047, 5.2-fold, HVO_B0144, 4.9-fold and HVO_B0198, 4.5-fold), and also one of the permeases (HVO_B0197, 12.7-fold) are up-regulated under iron starvation conditions. For each of these up-regulated proteins, the gene/operon is preceded by a DtxR transcription regulator binding motif I. None of the other fhu gene products was found to be regulated. One additional gene is preceded by Dtx binding motif I and the encoded protein is up-regulated under iron starvation: HVO_1228 (5.7-fold, hcpE, DUF5059 domain / halocyanin domain protein).

Of note is HVO_1464, which is strongly up-regulated (8.0-fold). It is a paralog of HVO_B0047 and putatively binds an iron-loaded siderophore, based on elaborate bioinformatic analysis (see below) (Leyn and Rodionov, 2015). The best SwissProt homolog, but with only low sequence similarity (a

BLASTp hit with 27% protein sequence identity covers only one-third of the proteins), is *B. subtilis* YfmC, a Fe^{3+}-citrate transporter. The ortholog from *Hbt. salinarum* (VNG_2549C, 46% protein sequence identity) belongs to the regulon of Idr1, one of the iron-responsive transcription regulators (Schmid *et al.*, 2011). It was proposed to be part of a potential hydroxamate siderophore transporter.

Supplementary Text S6: Analysis of the siderophore biosynthesis operon and the adjacently encoded ABC transporter binding protein

Cells responding to iron scarcity might be expected to increase the synthesis of siderophores for iron scavenging, and the siderophore biosynthesis cluster (HVO_B0046-HVO_B0041, dat-bdb-iucABDC) was recently implicated in the synthesis of schizokinen, a hydroxamate siderophore (Niessen and Soppa, 2020). All of the six corresponding gene products HVO_B0041 to HVO_B0046 were indeed detected in our proteome data, however HVO_B0045 (bdb) only qualitatively on the level of the spectral library. Surprisingly, expression changes for these gene products were somewhat inconsistent, with only HVO_B0044 (iucA) being significantly up-regulated under conditions of iron scarcity (i.e., 3.8-fold in the membrane fraction) and HVO_B0041 (iucC) being strongly down-regulated (10.4-fold). Interestingly, the neighbouring gene HVO_B0047 also showed significantly increased abundance under conditions of iron starvation (5.2-fold). This protein is a periplasmic substrate-binding protein of an ABC transporter, which putatively binds an iron-loaded siderophore, based on bioinformatic analysis (Leyn and Rodionov, 2015).

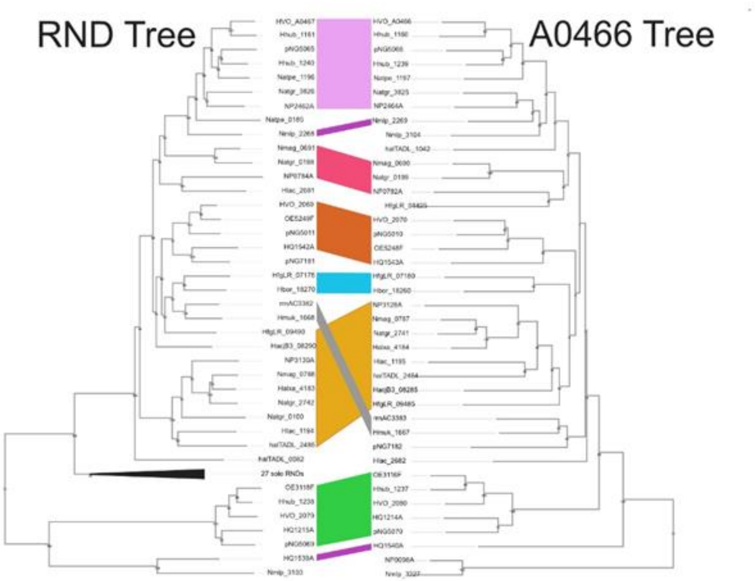

Supplementary Figure S3. Phylogenetic analysis of HVO_A0467 and HVO_A0466. Phylogenetic trees for HVO_A0467 and HVO_A0466 were computed separately as described in the Materials and Methods section, and are displayed side by side. A set of 27 RNDs transporters forming a monophyletic clade of which none is encoded adjacent to a HVO_A0466 homolog is collapsed ("27 solo RNDs"). Colored blocks highlight phylogenetic branches of either the HVO_A0467 or the tree HVO_A0466 tree when their adjacently encoded genes are also adjacent in the phylogenetic tree, but no necessarily in the same branch. The colored blocks represent protein groups 1 to 7 as also listed in Suppl.Table 6.

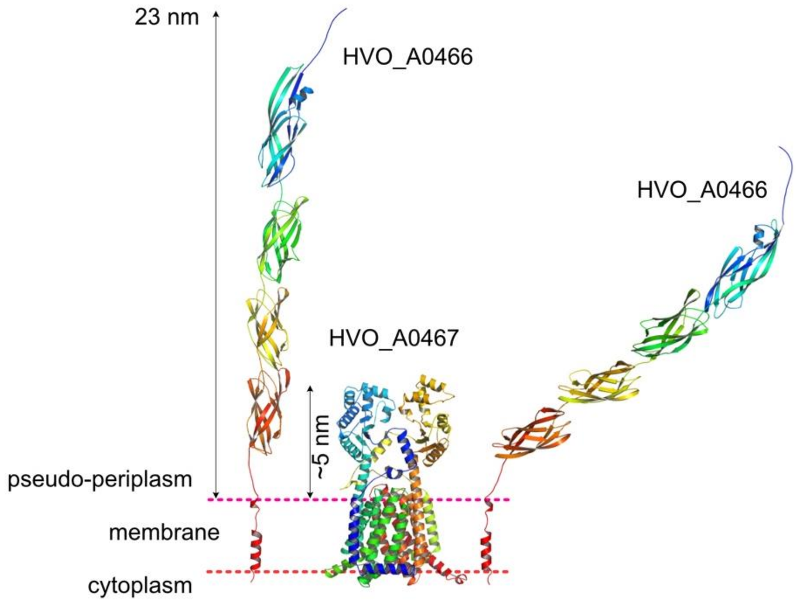

Supplementary Figure S4. The structure predicted for HVO_A0467 and HVO_A0466 by AlphaFold. The AlphaFold predicted 3D structures were downloaded as a pdb files from UniProtKB (date of accession May 24th, 2023) as described in the Materials and Methods section.

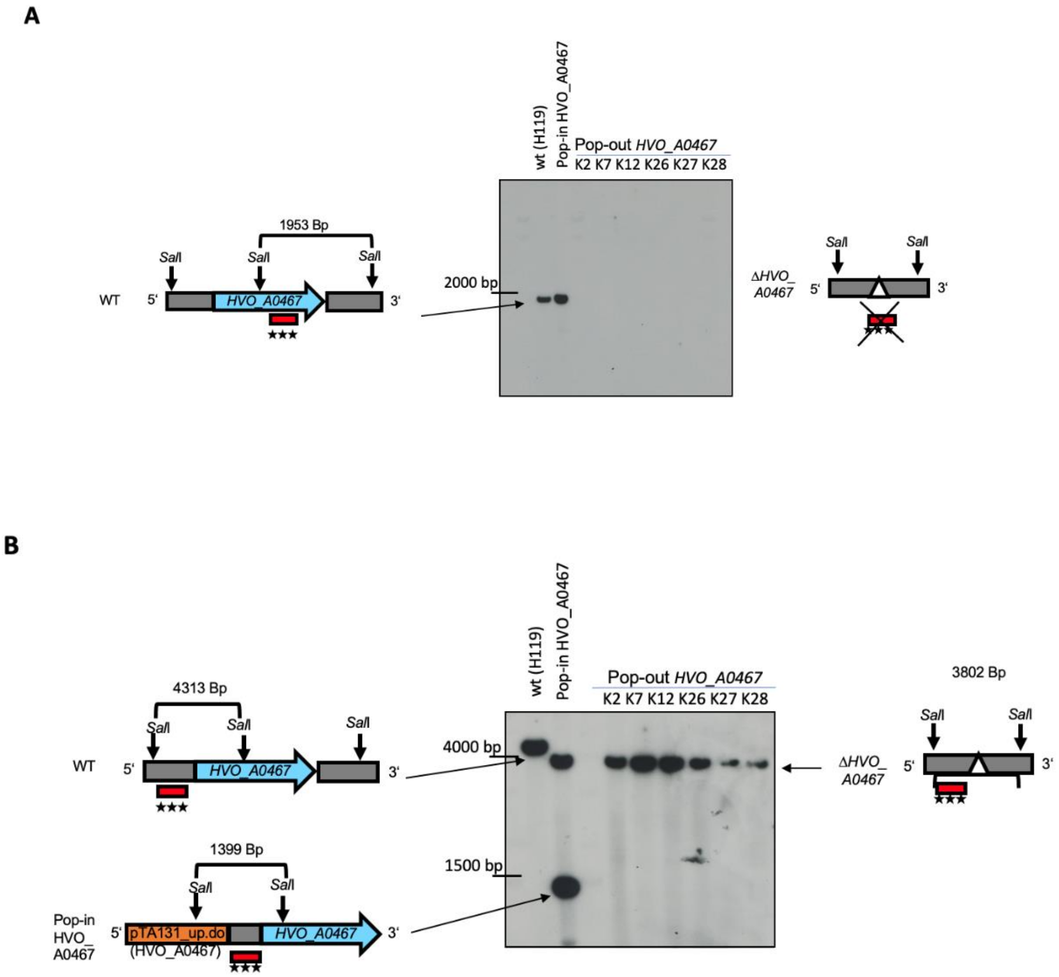

Supplementary Figure S5. Validation of deletion of HVO_A0467 by Southern blot. Genomic DNA was isolated from *Hfx. volcanii* H119, a pop-in clone and several potential deletion clones. The gDNA was digested with SalI and subsequently separated on a 0.8% agarose gel. After transfer to a nylon membrane, the membrane was hybridized with radioactive labelled PCR probes binding to HVO_A0467 (A) or in the upstream region of HVO_A0467 (B). A schematic representation of the binding sites of the probes (red) are shown schematically at the sides. Expected fragments are indicated with arrows.

References

Allers, T., Ngo, H.-P., Mevarech, M., Lloyd, R.G. (2004). Development of additional selectable markers for the halophilic archaeon Haloferax volcanii based on the leuB and trpA genes. *Appl. Environ. Microbiol.* 70:943-953. doi: 10.1128/AEM.70.2.943-953.2004.

Jevtic, Z., Stoll, B., Pfeiffer, F., Sharma, K., Urlaub, H., Marchfelder, A., and Lenz, C. (2019). The response of Haloferax volcanii to salt and temperature stress: a proteome study by label-free mass spectrometry. Proteomics 19, e1800491. doi: 10.1002/pmic.201800491.

Klein, C., Garcia-Rizo, C., Bisle, B, Scheffer, B., Zischka, H. Pfeiffer, F., et al. (2005) The membrane proteome of Halobacterium salinarum. *Proteomics* 5:180-97. doi: 10.1002/pmic.200400943.

Leyn, S.A., and Rodionov, D.A. (2015). Comparative genomics of DtxR family regulons for metal homeostasis in Archaea. *J. Bacteriol.* 197:451-458. doi: 10.1128/JB.02386-14.

Niessen, N., and Soppa, J. (2020). Regulated iron siderophore production of the halophilic archaeon Haloferax volcanii. *Biomolecules* 10:1072. doi: 10.3390/biom10071072.

Pfeiffer, F. and Oesterhelt, D. (2015) A manual curation strategy to improve genome annotation: application to a set of haloarchaeal genomes. *Life (Basel)* 5:1427-44. Doi: 10.3390/life5021427.

Schulze, S., Adams, Z., Cerletti, M., De Castro, R., Ferreira-Cerca, S., Fufezan, C., et al. (2020). The Archaeal Proteome Project advances knowledge about archaeal cell biology through comprehensive proteomics. *Nat. Commun.* 11, 3145. doi: 10.1038/s41467-020-16784-7.

Schmid, A.K., Pan, M., Sharma, K., and Baliga, N.S. (2011). Two transcription factors are necessary for iron homeostasis in a salt-dwelling archaeon. *Nucleic Acids Res*. 397, 2519-2533. doi: 10.1093/nar/gkq1211.
